# When absence becomes functional: lysine deserts in protein regulation and evolution

**DOI:** 10.3389/fmolb.2026.1959694

**Published:** 2026-10-12

**Authors:** Natalia A. Szulc

**Affiliations:** Laboratory of Protein Metabolism, International Institute of Molecular and Cell Biology in Warsaw, Warsaw, Poland

**Keywords:** lysine depletion, lysine desert, protein evolution, proteostasis, pupylation, ubiquitin-proteasome system (UPS), ubiquitylation (ubiquitination)

## Abstract

Lysine deserts–extended, continuous protein regions containing no lysine residues–occur across diverse proteomes, yet their definition, evolutionary origins and biological significance remain incompletely understood. In this Review, I examine the evidence linking local lysine depletion to protein regulation, with particular emphasis on ubiquitin- and proteasome-dependent pathways. Studies of toxin catalytic subunits and endogenous components of the ubiquitin-proteasome system show that introducing lysines at selected positions can alter ubiquitylation, protein turnover or function. These findings support a context-dependent model in which lysine deserts can limit the availability of ubiquitin acceptor sites within particular structural and enzymatic environments, rather than generally protecting entire proteins from modification or degradation. I discuss how lysine position, solvent accessibility, E3 ligase geometry and protein conformation determine the consequences of lysine introduction, and how N-terminal or non-lysine ubiquitylation as well as ubiquitin-independent proteolysis can bypass lysine depletion. I also consider the reported enrichment of lysine deserts in selected Actinobacteria and *Mycobacterium* phages, highlighting a testable Pup-proteasome adaptation hypothesis. Finally, I discuss potential functions beyond ubiquitylation and the methodological challenges of distinguishing selected lysine depletion from compositional bias or chance.

## 1 Introduction

Extended lysine-poor regions occur in bacterial and plant toxins, phage proteins and endogenous components of the ubiquitin system, raising the possibility that lysine depletion can shield proteins from lysine-directed ubiquitylation or degradation. In selected experimental systems, restoring lysines at particular positions alters ubiquitylation, protein abundance or function. These effects depend on molecular context, however, and do not by themselves explain why the regions are lysine-poor. How unusual, then, is such extensive lysine depletion?

Answering this question requires considering how genome composition, protein architecture, and ecology, among other factors, shape amino acid usage (Morimoto and Pietras, 2024). Lysine itself is not rare: it constitutes approximately 5% of residues in UniProtKB and 5.8% in its reviewed Swiss-Prot subset, corresponding to roughly one residue in every 17–20 positions (The UniProt Consortium, 2025; 2026a; 2026b). Under a deliberately simplified model in which residues are sampled independently and each position has a 5% probability of being lysine, a 300-residue sequence contains no lysine with an expected frequency of approximately 2 × 10^−7^. Real proteins exhibit regional compositional heterogeneity and dependencies between residues, but this simple calculation illustrates why extensive lysine depletion in an otherwise compositionally typical sequence warrants closer examination.

However, even a statistically unexpected absence does not by itself show that natural selection favored the elimination of an amino acid. Zhao and colleagues found that 14.1%–23.4% of annotated full-length proteins lacked at least one standard amino acid. These proteins were generally shorter than their respective proteome averages, and amino acid absence was inversely related to the background abundance of the missing residue, suggesting that many cases could arise without residue-specific selection. More suggestively, 25 pairs of human and mouse orthologs lacked lysine in both species–an early proteome-scale observation consistent with the possibility that lysine absence in some proteins is evolutionarily maintained (Zhao et al., 2008). These contrasting observations motivate a definition that distinguishes statistically unsurprising whole-protein absences from the unusually long lysine-depleted regions considered here.

The potential functional significance of these unusually long regions emerged from studies of toxins and E3 ubiquitin ligases, in which lysine depletion was experimentally linked to protein stability (Deeks et al., 2002; Fredrickson et al., 2013). Sharma and colleagues subsequently introduced the term *lysine desert* for a 398-residue lysine-free region in the yeast ubiquitin ligase Slx5 (Sharma et al., 2017). The term has since been applied more broadly, but no universal threshold exists. Kampmeyer et al. identified each protein’s longest uninterrupted lysine-free stretch and ranked proteins by its absolute length without imposing a universal cutoff, whereas Szulc et al. conducted proteome-wide screens using two operational criteria: a continuous lysine-free region of at least 150 residues or one spanning at least 50% of the sequence (Kampmeyer et al., 2023; Szulc et al., 2023). In this Review, the term lysine desert refers to an extended, continuous region of a protein sequence containing no lysine residues (Figure 1A). The term itself describes a sequence pattern and does not, by itself, imply that lysine depletion arose through natural selection.

**FIGURE 1:**
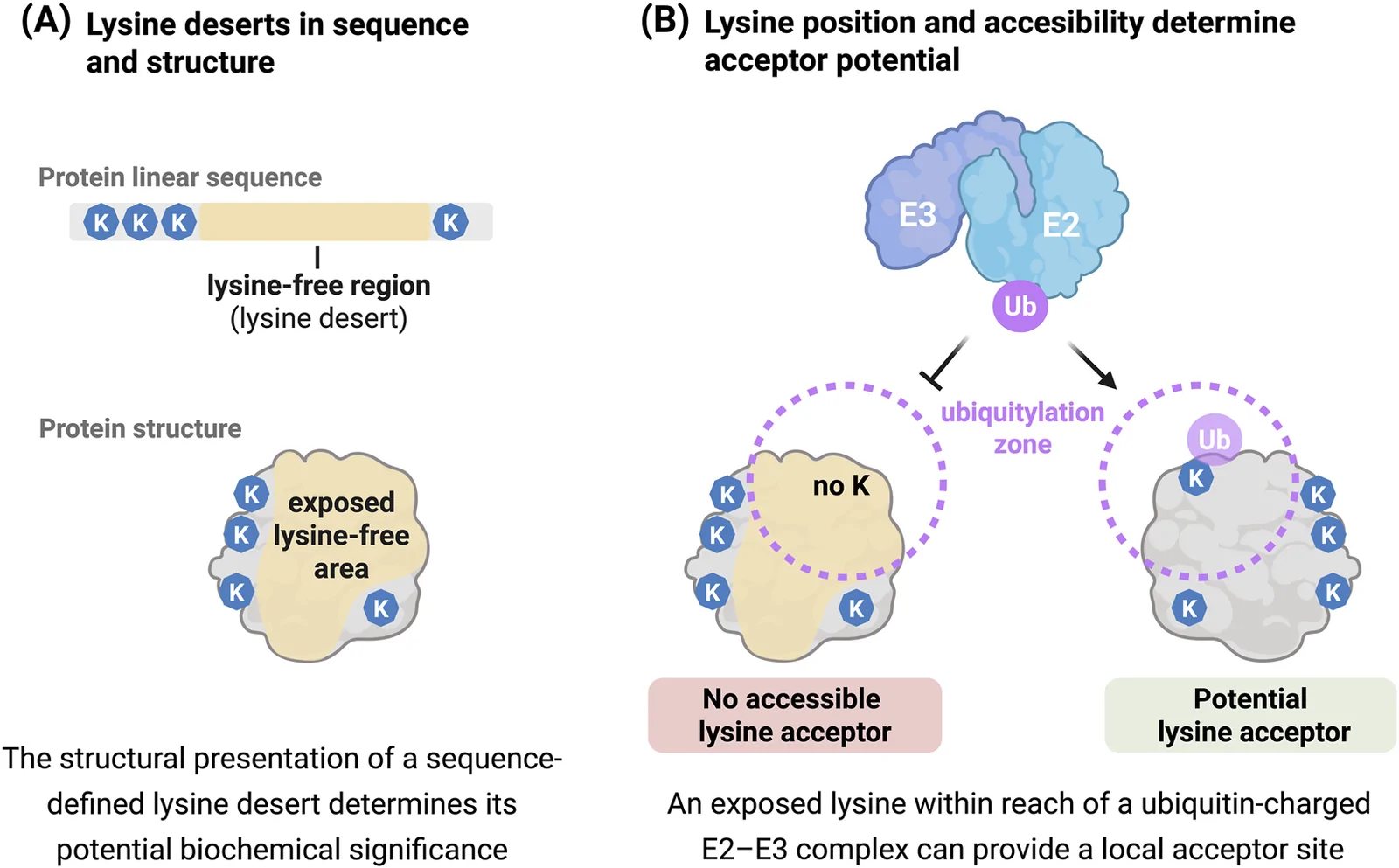
Potential influence of lysine deserts on local ubiquitylation. **(A)** Sequence and structural representations of a lysine desert. **(B)** Ubiquitin acceptor availability depends on whether a surface-exposed lysine lies within reach of a ubiquitin-charged E2–E3 complex. K, lysine; Ub, ubiquitin. Created in BioRender.com.

Absolute length and proportional sequence coverage capture different forms of lysine depletion. A long lysine-free region occupying only a small part of a multidomain protein may be confined to one domain or an intrinsically disordered region. By contrast, a shorter desert spanning most of a compact protein indicates that potential lysine acceptor sites are scarce throughout nearly the entire sequence. Neither measure, however, reveals how the desert is presented in three dimensions. After folding, a sequence-defined desert may form an exposed lysine-free surface, remain buried or become distributed across separate surfaces. Its biochemical significance therefore depends on structural accessibility and geometry relative to the relevant molecular machinery. Accordingly, no single cutoff is likely to be appropriate for all proteins and proteomes. Where possible, studies should report both absolute length and fractional coverage, describe the relevant domain, disorder and structural context, and establish whether their conclusions are robust to alternative thresholds.

Regardless of the operational definition used, lysine depletion can have important biochemical consequences. Lysine combines a positively charged side chain with an ε-amino group that serves as an acceptor for ubiquitin and ubiquitin-like modifiers and is also a site of methylation and of numerous acyl modifications (Hao et al., 2024). Depleting lysine from a protein region can therefore alter electrostatics and intermolecular interactions while simultaneously removing several routes of covalent regulation. The ubiquitin-proteasome system (UPS) provides the clearest mechanistic setting in which such depletion could influence protein fate. Ubiquitin is typically transferred through an E1–E2–E3 enzyme cascade and is most often joined to a substrate lysine through an isopeptide bond. Depending on context, ubiquitylation can regulate trafficking, signaling, DNA repair and other processes or promote proteasomal degradation (Dikic and Schulman, 2023).

Lysine availability can determine whether a conventional ubiquitin acceptor is present, but not every ubiquitylation event leads to proteasomal degradation. Productive degradation generally requires an accessible acceptor positioned within favorable E3–substrate geometry, a suitable ubiquitin signal, and an unstructured initiation segment that the 26S proteasome can engage to start substrate unfolding (Arkinson et al., 2025). A protein may therefore gain local protection without eliminating every lysine. Depletion of surface-exposed lysines favorably positioned to accept ubiquitin from the relevant E3 can reduce productive ubiquitylation within that region, creating local molecular insulation (Figure 1B). Such protection would not be absolute, because ubiquitin can sometimes be attached to the N-terminus or to non-lysine side chains, and proteins can also undergo ubiquitylation-independent proteasomal degradation (Pande et al., 2006; Shimizu et al., 2010; Scaglione et al., 2013; Dikic and Schulman, 2023).

Throughout this Review, local molecular insulation refers to the hypothesis that lysine deserts protect specific protein regions from ubiquitylation. This model predicts that introducing an accessible lysine into a lysine desert should result in ubiquitylation of the newly introduced residue, provided that the relevant ubiquitin-transfer machinery can access it. Whether this modification subsequently affects protein stability or function is a separate question. Conversely, failure to detect ubiquitylation argues against the molecular insulation model only when the relevant enzymes are known to be present and active; otherwise, a negative result remains inconclusive.

Studies of AB toxins and the endogenous E3 ligases San1 and Slx5–Slx8 provide the starting point for evaluating this model. I examine how these foundational experiments and subsequent studies of other UPS components support or qualify local protection from lysine-directed ubiquitylation. I then consider how alternative ubiquitylation and proteolytic pathways bypass lysine depletion, whether lysine desert enrichment in selected Actinobacteria and their phages supports a Pup-proteasome adaptation hypothesis, and which experimental limitations constrain mechanistic and evolutionary interpretation.

## 2 Restoring lysines creates a proteasome-linked liability in toxins

Early links between lysine scarcity and resistance to proteolysis emerged from studies of toxins that traffic through the endoplasmic reticulum (ER) and were proposed to exploit ER-associated degradation (ERAD) to reach the cytosol. London and Luongo found a much stronger Arg/Lys usage bias in the catalytic A domains of several AB toxins than in their receptor-binding B domains. The direction of this bias varied: individual catalytic domains showed nearly exclusive use of either arginine or lysine, and guanine–cytosine (GC) gene content alone did not adequately explain the domain-specific pattern (London and Luongo, 1989). Hazes and Read subsequently gave the lysine-poor subset a mechanistic interpretation. Focusing on ER-directed toxins including ricin, cholera toxin and pertussis toxin, they proposed that these proteins enter the cytosol by engaging components of ERAD. This route creates a potential problem for the toxin: proteins entering this pathway may be ubiquitylated and degraded by cytosolic proteasomes, whereas a toxin must persist long enough to act. Hazes and Read suggested that the exceptionally low lysine content of the catalytic A chains, a pattern now discussed in terms of lysine deserts, reduces opportunities for host-mediated ubiquitylation and may allow some dislocated toxin molecules to escape proteasomal destruction (Hazes and Read, 1997).

Targeted mutagenesis subsequently tested this degradation-avoidance hypothesis and showed that lysine introduction can create a proteasome-linked liability in ER-trafficking toxin subunits, although the completeness of the mechanistic evidence varies among studies. Ricin A chain (RTA) contains only two native lysines. Replacing both with arginine did not increase toxin potency, suggesting that these lysines do not normally serve as productive acceptors for degradation-linked ubiquitylation. By contrast, introducing four additional lysines reduced cytotoxicity by two orders of magnitude without detectably compromising catalytic activity, structure or intrinsic stability. Proteasome inhibition restored the potency of the lysine-rich RTA variant toward wild-type levels, and defective ubiquitin activation also alleviated this loss of cytotoxicity. Introducing four additional lysines into abrin A chain likewise caused a proteasome-sensitive loss of cytotoxicity (Deeks et al., 2002). These findings provide indirect support for protective role of lysine scarcity by showing that lysine introduction created a proteasome-sensitive loss of toxin activity without impairing catalytic activity or stability.

Experiments with cholera toxin A1 (CT A1) showed that retrotranslocation and cellular activity required neither internal lysines nor a free N-terminal α-amino group, as demonstrated using a lysine-free variant with a chemically blocked N-terminus. Introducing lysines at selected positions caused moderate, proteasome-sensitive attenuation of toxin function without evidence of altered folding (Rodighiero et al., 2002). A subsequent study examined how an unstructured N-terminal extension altered the fate of CT A1 after retrotranslocation. This extension impaired CT A1 refolding *in vitro* and promoted its rapid proteasomal degradation in the cytosol. Replacing both native lysines with arginines allowed the extended A1 chain to accumulate in the cytosol without requiring proteasome inhibition. Proteasome inhibition nevertheless further increased the activity of the N-terminally extended, lysine-free construct unless its N-terminal α-amino group was chemically blocked, suggesting that the N-terminus may provide an alternative ubiquitin acceptor site. Although CT A1 ubiquitylation was not detected directly, these findings implicated both the native lysines and the free N-terminal α-amino group in the proteasomal susceptibility created by the N-terminal extension (Wernick et al., 2010). CT A1 retrotranslocation was also sensitive to the activity of the deubiquitinase YOD1, which was proposed to act on ubiquitylated ERAD components rather than on CT A1 itself (Bernardi et al., 2013). Together, the CT A1 findings support a context-dependent form of molecular insulation in which lysine scarcity limits direct proteasomal susceptibility, while N-terminal context, protein folding and ubiquitin-regulated ERAD machinery continue to shape toxin fate.

A similar position-dependent pattern was observed for the naturally lysine-free S1 catalytic subunit of pertussis toxin (PT S1). The Arg→Lys variants tested retained wild-type-like enzymatic activity *in vitro* but showed reduced cellular activity, with the magnitude of the effect depending on the position and number of introduced lysines. A matched Arg→Ala control retained near-wild-type cellular activity, supporting an effect of lysine introduction rather than arginine loss. Proteasome inhibition substantially increased the cellular activity of variants containing more than one introduced lysine, with the greatest effect observed for the triple-site variant (Worthington and Carbonetti, 2007). These results therefore indirectly support a protective role for lysine scarcity by linking the position and number of introduced lysines to proteasome-sensitive loss of cellular activity without impairing enzymatic function.

Collectively, these toxin studies indicate that protection from degradation depends on local molecular context rather than lysine number alone. Lysine scarcity may therefore restrict lysine-directed ubiquitylation locally without conferring intrinsic, protein-wide resistance to degradation. However, ubiquitylation was not demonstrated for any of the toxin variants, and neither the implicated lysines nor the proposed N-terminus was directly identified as a ubiquitin acceptor. For RTA, abrin A chain and PT S1, degradation was inferred from proteasome-sensitive changes in toxin activity rather than measured directly. More generally, lysine depletion does not prevent ubiquitin-independent proteolysis. Isolated CT A1 and PT S1 are thermally unstable near physiological temperature, and purified 20S proteasomes degraded the unfolded subunits *in vitro* in the absence of ubiquitin-conjugation machinery (Pande et al., 2006; Massey et al., 2009). Proteasome engagement, however, need not be destructive: native, lysine-poor RTA was inefficiently degraded by the mammalian 26S proteasome *in vitro*, while the isolated 19S regulatory particle limited RTA aggregation and promoted recovery of its catalytic activity (Pietroni et al., 2013). Table 1 places these toxin findings alongside other lysine-restoration studies and summarizes their experimental approaches, outcomes, mechanistic evidence and principal limitations. Across the toxin systems examined here, lysine introduction is therefore best understood as creating a context-dependent, position- and folding-sensitive proteostatic liability.

**TABLE 1:** Experimental evidence for the molecular consequences of altering lysine availability in lysine-poor proteins and regions.

Protein	Native pattern	Perturbation	Outcome of perturbation	Mechanistic evidence and controls	Relevance to molecular insulation hypothesis	Principal limitations	References
Ricin A chain	Two native lysines	Lysine-free variant; double- and quadruple-site substitutions introducing lysine	Abundance/turnover: − Function: The lysine-introduced variants retained catalytic activity but showed a 25- to approximately 100-fold reduction in cytotoxicity, with the greatest reduction for the quadruple-site lysine variant	Modification/acceptor resolution: − Pathway perturbation: E1 inactivation and proteasome inhibition each alleviated the reduction in cytotoxicity of the quadruple-site lysine variant Specificity controls: The lysine-free variant did not alter toxin potency; a matched quadruple-site arginine variant retained near-native cytotoxicity; the lysine-introduced variants retained intrinsic stability and native-like structure	Indirect support: Lysine introduction caused an E1- and proteasome-sensitive reduction in cytotoxicity	Protein degradation and ubiquitylation were not measured directly; the introduced lysines were not identified as ubiquitin acceptors	Deeks et al., 2002
Abrin A chain	Three native lysines	Lysine-free variant; double- and quadruple-site substitutions introducing lysine	Abundance/turnover: − Function: The lysine-introduced variants retained catalytic activity but showed reduced cytotoxicity, with a greater reduction for the quadruple-site lysine variant	Modification/acceptor resolution: − Pathway perturbation: Proteasome inhibition alleviated the reduction in cytotoxicity of the quadruple-site lysine variant Specificity controls: The lysine-free variant did not alter toxin potency; the lysine-introduced variants retained intrinsic stability and normal association with and dissociation from the B chain	Indirect support: Lysine introduction caused a proteasome-sensitive reduction in cytotoxicity	Protein degradation and ubiquitylation were not measured directly; the introduced lysines were not identified as ubiquitin acceptors	Deeks et al., 2002
Cholera toxin A1 subunit	Two native lysines	Lysine-free variant; single- and double-site substitutions introducing lysine; chemical blockade of the N-terminal α-amino group; addition of two different unstructured N-terminal extensions	Abundance/turnover: In one N-terminally extended construct, the A1 chain accumulated in the cytosol only after proteasome inhibition, whereas removal of both native lysines permitted its accumulation without inhibition Function: A lysine-free variant with a chemically blocked N-terminus retained cellular activity; introduction of one or two lysines moderately reduced toxin activity; both N-terminal extensions caused strong reductions in cellular activity	Modification/acceptor resolution: Ubiquitylation was not detected Pathway perturbation: Proteasome inhibition restored the cellular activity of the lysine-introduced variants and partially restored that of both N-terminally extended constructs Specificity controls: The lysine-introduced variants showed no detectable folding defects; both N-terminal extensions reduced toxin activity, and proteolytic removal of either restored activity	Indirect support: Lysine introduction reduced toxin activity in a proteasome-sensitive manner, whereas removing both native lysines protected an N-terminally extended construct from proteasome-sensitive loss	Ubiquitylation was not detected; the implicated lysines and N-terminal α-amino group were not identified as ubiquitin acceptors; one N-terminal extension also impaired refolding *in vitro*, preventing attribution of its effects solely to altered ubiquitin acceptor availability	Rodighiero et al., 2002; Wernick et al., 2010
Pertussis toxin S1 subunit	Lysine-free	Single-, double- and triple-site Arg→Lys variants	Abundance/turnover: − Function: The lysine variants retained wild-type-like enzymatic activity *in vitro* but showed position- and number-dependent reductions in cellular activity	Modification/acceptor resolution: Ubiquitylation was not detected Pathway perturbation: Proteasome inhibition substantially increased the cellular activity of variants containing more than one introduced lysine Specificity controls: A matched double-alanine control retained near-wild-type cellular activity; the lysine variants retained enzymatic activity *in vitro*	Indirect support: Lysine introduction caused a position- and number-dependent, proteasome-sensitive reduction in cellular toxin activity	Protein degradation was not measured directly; ubiquitylation was not detected; the introduced lysines were not identified as ubiquitin acceptors	Worthington and Carbonetti, 2007
*S. cerevisiae* San1	13 native lysines clustered in or near the RING domain; disordered N- and C-terminal substrate-binding regions are lysine-free	17 individual Arg/Asn→Lys substitutions; two representative substitutions tested as sole lysines in otherwise lysine-free San1; two individual Arg→Lys substitutions in the *S. pombe* ortholog, tested in *S. cerevisiae*	Abundance/turnover: All 17 *S. cerevisiae* lysine variants were rapidly degraded: 11 predominantly *in cis*, three predominantly *in trans* and three predominantly through a San1-independent route; RING inactivation increased the steady-state abundance of all 17 variants; representative sole-lysine constructs and the *S. pombe* variants underwent RING-dependent degradation Function: −	Modification/acceptor resolution: Increased protein-level ubiquitylation relative to wild-type San1 was demonstrated for two representative lysine variants; their ubiquitylation was RING-dependent and persisted in the corresponding sole-lysine constructs Pathway perturbation: RING inactivation and the presence or absence of endogenous San1 distinguished predominantly *in cis*, *in trans* and San1-independent degradation Specificity controls: Matched arginine or asparagine controls at the same 17 positions did not destabilize San1; selected lysine variants retained substrate binding; lysine-free San1 retained function after restoration of its nuclear localization	Support: Introducing a single lysine at each of 17 positions across the two lysine-free regions caused rapid degradation, with a predominantly *in cis* mechanism in 11 variants and a contribution from RING activity in all 17; representative lysine variants, including their sole-lysine versions, also showed RING-dependent protein-level ubiquitylation	The sole-lysine constructs strongly implicated the introduced lysines as ubiquitin acceptors, but the ubiquitin–lysine junctions were not mapped directly; proteasome dependence was not tested	Fredrickson et al., 2013
*S. cerevisiae* Slx5	398-aa lysine desert	Ten distinct single-site substitutions introducing lysine, including two variants identified in a suppressor screen and a positional series at 50-residue intervals; deletion of just over half of the lysine desert	Abundance/turnover: The two screen-derived lysine variants showed markedly reduced cellular abundance, which increased following proteasome inhibition and was restored to approximately wild-type levels by RING inactivation Function: The screen-derived variants were hypomorphic, with the extent of impairment varying among genetic backgrounds; the effects of lysine introduction varied by position in suppression and complementation assays; deletion of part of the lysine desert produced a well-expressed protein with impaired function	Modification/acceptor resolution: Purified Slx5–Slx8 complexes containing either screen-derived lysine variant showed increased Slx5 autoubiquitylation *in vitro* Pathway perturbation: Proteasome inhibition increased the abundance of the two screen-derived variants, while RING inactivation restored approximately wild-type levels; wild-type Slx5 did not efficiently destabilize their RING-inactive versions *in trans* Specificity controls: Three matched arginine controls did not reproduce the suppressor phenotype; those corresponding to the two screen-derived lysine variants retained near-wild-type abundance and did not enhance Slx5 autoubiquitylation *in vitro*	Support: Introducing lysine at the two screen-derived positions increased Slx5 autoubiquitylation *in vitro* and caused RING- and proteasome-dependent loss of abundance	The introduced lysines were not identified as ubiquitin acceptors; enhanced Slx5 autoubiquitylation was demonstrated with purified complexes but not directly in cells	Sharma et al., 2017
*S. cerevisiae* Hrd1	Two predicted disordered cytosolic regions devoid of lysine and cysteine	Deep mutational scanning; selected single-site substitutions introducing lysine or cysteine across both depleted regions; a double-lysine variant; replacement of the depleted regions with linkers	Abundance/turnover: Most tested lysine and cysteine variants showed reduced cellular abundance Function: Lysine and cysteine substitutions in either depleted region impaired degradation of luminal and membrane endoplasmic reticulum-associated degradation substrates; the double-lysine variant retained autoubiquitylation, substrate ubiquitylation and retrotranslocation activities comparable to wild-type Hrd1 in a reconstituted system; linker replacements showed that the two depleted regions contribute differently to Hrd1 autoubiquitylation and substrate retrotranslocation	Modification/acceptor resolution: Autoubiquitylation was demonstrated *in vitro* for single- and double-lysine variants; the introduced lysine in the single-lysine variant was mapped as a ubiquitin acceptor by mass spectrometry Pathway perturbation: Catalytic inactivation prevented the reduction in abundance of selected lysine and cysteine variants Specificity controls: The depleted regions tolerated many other amino-acid substitutions; retention of activity by the double-lysine variant argues against direct impairment of Hrd1 by lysine introduction	Support: An introduced lysine was directly mapped as a ubiquitin acceptor, and catalytic inactivation prevented the reduction in abundance of selected lysine and cysteine variants	Only one introduced lysine was mapped as a ubiquitin acceptor in a single experiment; cellular ubiquitylation and modification of the cysteine variants were not demonstrated directly; turnover kinetics and proteasome dependence were not tested	Peterson et al., 2023
Human RAD23A	198-aa lysine desert	Multi- and single-site Arg→Lys variants	Abundance/turnover: The multi-site lysine variant showed no detectable reduction in steady-state abundance Function: The multi-site lysine variant complemented the UV-sensitive phenotype of *S. pombe* cells lacking their endogenous RAD23A ortholog less effectively than wild-type RAD23A	Modification/acceptor resolution: Denaturing enrichment demonstrated increased protein-level ubiquitylation of the multi-site lysine variant; two introduced lysines were mapped as ubiquitin acceptors by mass spectrometry Pathway perturbation: Overexpressed active, but not catalytically inactive, E6AP enhanced ubiquitylation of the multi-site lysine variant Specificity controls: The matched multi-site Arg→Gln control did not reproduce the increased ubiquitylation; individual lysine substitutions showed positional dependence	Support: Lysine introduction created directly mapped ubiquitin acceptors and increased protein-level ubiquitylation without reducing abundance, but was associated with impaired complementation	Functional complementation was tested in a heterologous system; ubiquitylation of the introduced lysines was not shown to cause the complementation defect, and indirect effects on protein folding, structure or localization could not be excluded	Kampmeyer et al., 2023
Human UBQLN1	482-aa lysine desert; lysines concentrated in the N-terminal ubiquitin-like domain	Multi- and single-site Arg→Lys variants	Abundance/turnover: The multi-site lysine variant showed reduced abundance that increased following proteasome inhibition Function: Neither the multi-site lysine variant nor the matched Arg→Gln control formed detectable cytosolic speckles	Modification/acceptor resolution: Denaturing enrichment demonstrated increased protein-level ubiquitylation of the multi-site lysine variant; two introduced lysines were mapped as ubiquitin acceptors by mass spectrometry Pathway perturbation: Proteasome inhibition increased the abundance of the multi-site lysine variant Specificity controls: The matched multi-site Arg→Gln control did not reproduce the increased ubiquitylation; individual lysine substitutions showed positional dependence	Support: Lysine introduction created directly mapped ubiquitin acceptors, increased protein-level ubiquitylation and caused a proteasome-sensitive reduction in abundance	Turnover kinetics were not measured; the loss of cytosolic speckles was not specific to lysine introduction and could reflect arginine loss, while reduced abundance may also have contributed to this phenotype	Kampmeyer et al., 2023
Human UBQLN2	521-aa lysine desert; lysines concentrated in the N-terminal ubiquitin-like domain	Double- and multi-site Arg→Lys variants	Abundance/turnover: The multi-site lysine variant showed reduced abundance that increased following proteasome inhibition or in E6AP-deficient cells Function: The multi-site lysine variant showed strongly reduced cytosolic puncta formation, which was largely restored in E6AP-deficient cells	Modification/acceptor resolution: Denaturing enrichment demonstrated increased protein-level ubiquitylation of both lysine variants; mass spectrometry identified six native lysines as ubiquitin acceptors Pathway perturbation: Proteasome inhibition and E6AP disruption increased the abundance of the multi-site lysine variant; ubiquitylation of wild-type UBQLN2 was unaffected by E6AP disruption Specificity controls: The matched multi-site Arg→Gln control did not behave like wild-type UBQLN2: it appeared more abundant, showed reduced ubiquitylation and had a broader cytosolic distribution	Support: Lysine introduction increased protein-level ubiquitylation and caused an E6AP- and proteasome-sensitive reduction in abundance	The introduced lysines were not identified as ubiquitin acceptors; turnover kinetics were not measured; the multi-site Arg→Gln control was not wild-type-like, complicating attribution of the observed effects solely to lysine introduction; the puncta phenotype could not be separated from changes in abundance or possible effects of arginine replacement on phase separation	Kampmeyer et al., 2023; Grønbæk-Thygesen et al., 2026
Human BAG6	950-aa lysine desert	Multi- and single-site Arg→Lys variants	Abundance/turnover: The multi-site lysine variant showed reduced abundance that increased following proteasome inhibition Function: −	Modification/acceptor resolution: Denaturing enrichment demonstrated increased protein-level ubiquitylation of the multi-site lysine variant Pathway perturbation: Overexpressed active, but not catalytically inactive, RNF126 enhanced ubiquitylation of the multi-site lysine variant; proteasome inhibition increased its abundance Specificity controls: The matched multi-site Arg→Gln control did not reproduce the increased ubiquitylation; individual lysine substitutions showed positional dependence	Support: Lysine introduction increased protein-level ubiquitylation, which was further enhanced by active RNF126, and caused proteasome-sensitive loss of abundance	The introduced lysines were not identified as ubiquitin acceptors; turnover kinetics were not measured	Kampmeyer et al., 2023
Human RNF115	173-aa lysine desert	Triple Arg→Lys variant	Abundance/turnover: − Function: −	Modification/acceptor resolution: Denaturing enrichment demonstrated increased protein-level ubiquitylation of the lysine variant Pathway perturbation: − Specificity controls: −	Limited support: Lysine introduction increased protein-level ubiquitylation	The introduced lysines were not identified as ubiquitin acceptors; no matched non-lysine control was tested; abundance, turnover kinetics and proteasome dependence were not tested	Kampmeyer et al., 2023
Human PSMF1/PI31	123-aa lysine desert	Single Arg→Lys variant	Abundance/turnover: − Function: −	Modification/acceptor resolution: No detectable increase in protein-level ubiquitylation of the lysine variant Pathway perturbation: − Specificity controls: −	No support under the conditions tested: One introduced lysine produced no detectable increase in protein-level ubiquitylation; the relevance of other positions remains unresolved	Only one lysine position and one experimental context were examined; abundance, turnover kinetics, proteasome dependence and function were not tested	Kampmeyer et al., 2023
Human VHL	Three native lysines; cullin-RING ligase assembly may restrict access to one or two	Lysine-free variant; two distinct triple Arg→Lys variants	Abundance/turnover: Wild-type and lysine-free variants showed similar basal turnover and accumulated following proteasome inhibition; both lysine-introduced variants showed reduced stability Function: −	Modification/acceptor resolution: NanoBRET detected comparable ubiquitin-proximity signals for wild-type and lysine-free variants, which were reduced by E1 inhibition Pathway perturbation: Proteasome, E1 and neddylation inhibition each attenuated the destabilization of the lysine-introduced variants; E1 inhibition stabilized both wild-type and lysine-free variant, whereas neddylation inhibition stabilized only wild-type protein Specificity controls: Both lysine-introduced variants showed reduced stability; blocking the principal VHL substrate-binding site did not reduce the NanoBRET signal	Indirect support: Lysine introduction caused a neddylation-, E1- and proteasome-sensitive reduction in stability	Increased ubiquitylation of the lysine-introduced variants was not demonstrated; NanoBRET did not establish covalent VHL modification or identify ubiquitin acceptor sites; matched non-lysine substitutions were not tested	Szulc et al., 2023
Human SOCS1	One native lysine, predicted to be buried	Lysine-free variant; three distinct triple Arg→Lys variants	Abundance/turnover: Wild-type and lysine-free variants showed similar basal turnover and accumulated following proteasome inhibition; none of the lysine-introduced variants showed a detectable change in stability Function: −	Modification/acceptor resolution: NanoBRET detected comparable ubiquitin-proximity signals for wild-type and lysine-free variants, which were reduced by E1 inhibition Pathway perturbation: Proteasome inhibition stabilized wild-type, lysine-free and lysine-introduced variants; E1 and neddylation inhibition did not stabilize wild-type or lysine-free SOCS1 Specificity controls: None of the lysine-introduced variants showed a detectable stability effect	No support under the conditions tested: Lysine introduction did not detectably affect stability, and proteasome-sensitive but E1-insensitive turnover suggested that lysine availability was not rate-limiting for degradation	NanoBRET did not establish covalent SOCS1 modification or identify ubiquitin acceptor sites; no substrate-binding inhibitor was available to assess whether ubiquitylated SOCS1-bound proteins contributed to the signal; matched non-lysine substitutions were not tested; protein function was not assessed	Szulc et al., 2023
*C. elegans* EEL-1	Two lysine desert regions encompassing an acidic intrinsically disordered region and three ubiquitin-binding motifs	Three single-site substitutions introducing lysine (two screen-derived and one arbitrarily positioned); lysine-containing sequence and tag insertions	Abundance/turnover: Two screen-derived lysine substitutions strongly reduced endogenous HA-tagged EEL-1 abundance Function: All three lysine substitutions increased accumulation of a ubiquitin-fusion degradation reporter and caused position- and age-dependent vulval rupture; lysine-bearing sequence and tag insertions produced similar phenotypes; a lysine beside the binding tag weakened the reduction in abundance of a tethered fluorescent substrate	Modification/acceptor resolution: − Pathway perturbation: HECT-domain active-site inactivation increased the abundance of one tested screen-derived lysine variant and wild-type protein Specificity controls: Matched alanine or arginine substitutions and lysine-free insertions did not reproduce the lysine-associated phenotypes; recruitment of another tag-binding protein beside the ectopic lysine reduced ubiquitin-fusion degradation reporter accumulation and vulval rupture	Indirect support: Lysine introduction impaired protein function and reduced abundance, while ligase inactivation alleviated the loss of abundance; recruitment of a tag-binding protein beside an introduced lysine also reduced ubiquitin-fusion degradation reporter accumulation and improved vulval integrity	Ubiquitylation and modification of the introduced lysines were not demonstrated; turnover kinetics and proteasome dependence were not tested; neither ubiquitylation of the tethered substrate nor abundance of the lysine-bearing EEL-1 tethering construct was examined; Most functional phenotypes were examined in animals with a sensitized *skn-1a* genetic background	Yanagi et al., 2026

**−** The indicated outcome, mechanistic test or control was not assessed in the cited study; experimentally tested negative results are stated explicitly.

## 3 Lysine restoration reveals context-dependent molecular insulation within the ubiquitin system

The toxin studies raise a broader question: do endogenous components of the ubiquitin system face an analogous molecular constraint when their own surfaces are repeatedly positioned near ubiquitin-transfer machinery? E3 ubiquitin ligases, ubiquitin receptors, substrate adaptors and proteasomal shuttle factors interact with activated ubiquitin, E2 enzymes, substrates or the proteasome, potentially exposing selected regions to unintended ubiquitylation. Whether local lysine depletion reduces such modification while preserving ubiquitin-dependent regulation elsewhere in the protein, and whether it evolved for this purpose, remains an open question.

One of the first experimental demonstrations that lysine depletion can protect an endogenous protein came from the yeast nuclear quality control E3 ligase San1 (Fredrickson et al., 2013). San1 contains a central Really Interesting New Gene (RING) domain, the catalytic module that recruits a ubiquitin-charged E2 enzyme and facilitates ubiquitin transfer, flanked by flexible substrate-binding regions. These regions contain no lysines, whereas the protein’s few lysines are concentrated in or near the RING domain. Introducing a single lysine at any of 17 positions tested across the lysine-depleted regions caused rapid degradation, whereas matched arginine or asparagine substitutions did not. Using RING-inactive variants and cells with or without endogenous San1, the authors distinguished three predominant degradation routes: 11 variants were degraded mainly through San1-dependent autoubiquitylation *in cis*, three mainly through San1-dependent autoubiquitylation *in trans*, and three mainly through a San1-independent mechanism. RING inactivation nevertheless increased the steady-state abundance of all tested variants, indicating that autoubiquitylation *in cis* contributed to their degradation to varying degrees. In two representative variants, replacement of all native San1 lysines with arginine left the introduced residue as the sole lysine; both constructs were ubiquitylated and underwent RING-dependent degradation. This sole-lysine design strongly implicates the introduced residues as ubiquitin acceptors, although the attachment sites were not mapped directly and the study did not test whether degradation was blocked by proteasome inhibition. The broader organization of disordered, lysine-depleted regions was conserved across divergent fungal orthologs, and lysine introduction destabilized the *Schizosaccharomyces pombe* ortholog when expressed in *Saccharomyces cerevisiae* (Fredrickson et al., 2013). San1 therefore provides strong mechanistic support for molecular insulation, in which lysine depletion within flexible substrate-binding regions limits self-targeted degradation by the ligase’s own ubiquitin-transfer activity.

The yeast small ubiquitin-related modifier (SUMO)-targeted ligase Slx5–Slx8 provided independent genetic and biochemical evidence that a lysine desert can limit self-targeted degradation (Sharma et al., 2017). A synthetic-lethality screen recovered two independent suppressor alleles, each introducing a single lysine into the 398-residue Slx5 lysine desert. A third, engineered lysine substitution produced a similar suppressor phenotype, whereas the corresponding arginine substitutions did not. The two screen-derived variants were hypomorphic, with the severity of their functional impairment varying among genetic backgrounds. Purified Slx5–Slx8 complexes containing either variant showed increased Slx5 autoubiquitylation *in vitro*, implicating the introduced lysines as acceptors, although conjugation to these residues and enhanced Slx5 autoubiquitylation in cells were not demonstrated directly. Both variants showed markedly reduced cellular abundance, which increased after proteasome inhibition; inactivation of the Slx5 RING domain restored their abundance to approximately wild-type levels. The RING-inactive variants remained stable when coexpressed with wild-type Slx5, indicating that they were not efficiently targeted *in trans* and supporting predominantly *in cis* autoubiquitylation followed by proteasomal degradation. Wild-type Slx5 abundance also increased after proteasome inhibition or RING inactivation, indicating that the native lysine desert limits rather than eliminates self-targeted degradation. The lysine desert also contributed to Slx5–Slx8 function: deleting just over half of it produced a well-expressed protein that retained partial function but complemented the growth defect less efficiently than wild-type Slx5. Sequence comparisons identified the extended lysine desert principally in large fungal Slx5 homologs; smaller fungal homologs and mammalian RNF4 lack an equivalently long domain, although some contain proportionally extensive lysine-free regions of uncertain functional significance (Sharma et al., 2017). Taken together, the Slx5 findings support a model in which local lysine depletion limits excessive *in cis* autoubiquitylation and proteasomal loss within a functionally important region.

The yeast ERAD ligase Hrd1 provides a further example of local acceptor depletion potentially protecting an endogenous E3 ligase from deleterious autoubiquitylation (Peterson et al., 2023). Deep mutational scanning identified two predicted disordered cytosolic regions that tolerated many amino-acid substitutions but were selectively intolerant of lysine and cysteine substitutions. Introducing lysine or cysteine at selected positions in either region generally reduced Hrd1 abundance and impaired its ability to degrade ERAD substrates. Catalytic inactivation prevented the reduction in abundance of the tested variants, supporting the interpretation that their destabilization resulted from Hrd1 autoubiquitylation. Following *in vitro* autoubiquitylation, mass spectrometry directly identified one introduced lysine as a ubiquitin acceptor, although acceptor-site identification was performed in a single replicate. A double variant containing one introduced lysine in each depleted region nevertheless retained autoubiquitylation, substrate ubiquitylation and retrotranslocation activities comparable to wild-type Hrd1 in a reconstituted system. This finding argues against lysine introduction into these regions necessarily impairing Hrd1 activity directly and instead supports reduced cellular abundance as the main explanation for the ERAD defects. However, turnover kinetics and proteasome dependence were not tested, and the reconstituted assays contained Hrd1 without other proteins that can associate with it. Predicted disordered cytosolic regions depleted of lysine and cysteine were also identified in Hrd1 homologs from fungi to mammals, although their lengths and positions varied and the functional significance of this recurring pattern was not tested (Peterson et al., 2023). Hrd1 therefore extends the molecular insulation model beyond lysine depletion alone, suggesting that local exclusion of lysine and cysteine may preserve ligase abundance by limiting autoubiquitylation-associated loss within functionally important disordered regions.

Subsequent sequence-based surveys and targeted lysine-restoration experiments examined whether analogous protection occurs in other UPS components. A San1-inspired bioinformatic analysis identified lysine-poor, often disordered regions in additional yeast and human E3 ligases and in BAG6, thereby expanding the set of candidate examples without experimentally testing whether these regions share a protective mechanism (Boomsma et al., 2016). A subsequent proteome-scale screen identified long lysine-free regions in hundreds of human proteins and selected six UPS-related proteins for experimental analysis: RAD23A, UBQLN1, UBQLN2, BAG6, RNF115 and PSMF1 (Kampmeyer et al., 2023). Arg→Lys variants showed increased protein-level ubiquitylation in five of the six proteins, with PSMF1 as the exception; matched Arg→Gln controls tested for RAD23A, UBQLN1 and BAG6 did not reproduce this increase. Testing individual Arg→Lys substitutions in RAD23A, UBQLN1 and BAG6 showed that some positions increased protein-level ubiquitylation whereas others had no detectable effect. In a single mass-spectrometry experiment, introduced lysines were directly identified as ubiquitin acceptor sites in both RAD23A and UBQLN1. Overexpression of E6AP and RNF126, but not their catalytically inactive variants, enhanced ubiquitylation of lysine-restored RAD23A and BAG6, respectively, demonstrating that these E3s can promote the modification without establishing their necessity or exclusivity under endogenous conditions. To test whether increased ubiquitylation led to proteasomal degradation, the authors examined protein abundance in stable cell lines. The UBQLN1 and BAG6 Arg→Lys variants were less abundant and stabilized by proteasome inhibition, whereas RAD23A abundance was largely unaffected. This contrasting result prompted the authors to examine whether lysine restoration instead affected RAD23A function. The Arg→Lys variant complemented the UV-sensitive phenotype of *S. pombe* cells lacking their endogenous RAD23A ortholog less effectively than wild-type RAD23A. RAD23A therefore provides direct evidence that restored lysines can become ubiquitin acceptors, together with an associated functional phenotype, but does not support protection from degradation. Whether ubiquitylation of the introduced lysines caused the complementation defect remains unresolved because indirect effects on protein folding, structure or localization could not be excluded. For PSMF1, only one Arg→Lys substitution was tested, and downstream outcomes were not examined (Kampmeyer et al., 2023), leaving the relevance of its lysine desert to local protection unresolved. Overall, the study supports a protective role for lysine depletion beyond E3 ligases, with effects depending on the protein and position examined.

A follow-up study examined human UBQLN2 in greater detail, asking how the regional availability of lysines influences its ubiquitylation, turnover and subcellular localization (Grønbæk-Thygesen et al., 2026). A multi-site Arg→Lys variant in the lysine-depleted region showed greater protein-level ubiquitylation than wild-type UBQLN2, although the introduced lysines were not mapped as ubiquitin acceptors. The variant was also markedly less abundant, but its level increased following proteasome inhibition and in E6AP-deficient cells, consistent with E6AP-dependent proteasomal degradation. Because UBQLN2 normally forms cytosolic puncta, the authors also checked whether lysine introduction altered this localization pattern. The Arg→Lys variant showed strongly reduced puncta formation, which was largely restored in E6AP-deficient cells, although the puncta were smaller and fewer per cell. This phenotype cannot be separated from changes in protein abundance, however, because E6AP disruption also restored the level of this variant, while replacing arginine with lysine may itself reduce UBQLN2 propensity for phase separation. The matched Arg→Gln variant further complicates this interpretation because, compared to wild-type UBQLN2, it showed reduced ubiquitylation, appeared more abundant and displayed a broader cytosolic distribution, indicating that substitutions within this region can affect UBQLN2 through mechanisms other than the creation of ubiquitin acceptor sites. For comparison, mass spectrometry of overexpressed wild-type UBQLN2 identified six lysines within its N-terminal ubiquitin-like domain as ubiquitin acceptors, while protein-level ubiquitylation of the wild-type protein was unaffected by E6AP disruption (Grønbæk-Thygesen et al., 2026). Together, these results are consistent with a protective role for the UBQLN2 lysine desert: lysines introduced into this region sensitized UBQLN2 to E6AP-dependent proteasomal degradation, whereas wild-type protein-level ubiquitylation was unaffected by E6AP disruption under the tested conditions.

Lysine deserts have also been examined across eukaryotic proteomes and among modular cullin-RING ligases (CRLs), multiprotein E3 complexes activated by neddylation–the covalent attachment of the ubiquitin-like protein NEDD8 to the cullin scaffold (Harper and Schulman, 2021). Using both an absolute (≥150 residues) and a relative (≥50% of the sequence) lysine desert definition, Szulc and colleagues found the same broad trend: the proteome-wide prevalence of lysine deserts was greater in mammalian than fungal taxa (Figure 2A), and an analysis counting only lysine deserts conserved among orthologs produced the same pattern. The study also defined a lysine cluster as a region spanning no more than 20% of a protein’s sequence and containing a number of lysine residues at least equal to the integer obtained by rounding down 80% of the protein’s total lysine count, considering only proteins with at least two lysines (Figure 2B). Across the five selected eukaryotic proteomes (yeast, worm, fly, mouse and human), 31%–49% of proteins meeting the relative lysine desert criterion also met the lysine cluster definition (Figure 2C). Thus, an extended lysine-free region can coexist with concentration of most remaining lysines within a short sequence segment, suggesting that lysine availability is regionally partitioned in some proteins. Conserved eukaryotic proteostasis factors were prominent among proteins displaying this architecture. UBQLN1 and UBQLN2, discussed above, provide notable examples in which a long lysine desert co-occurs with concentration of the remaining lysines within the N-terminal domain. The analysis also extended to bacterial and phage proteomes, where a higher prevalence of lysine desert proteins was reported in selected Actinobacteria and *Mycobacterium* phages (Szulc et al., 2023), as discussed in Section 5.

**FIGURE 2:**
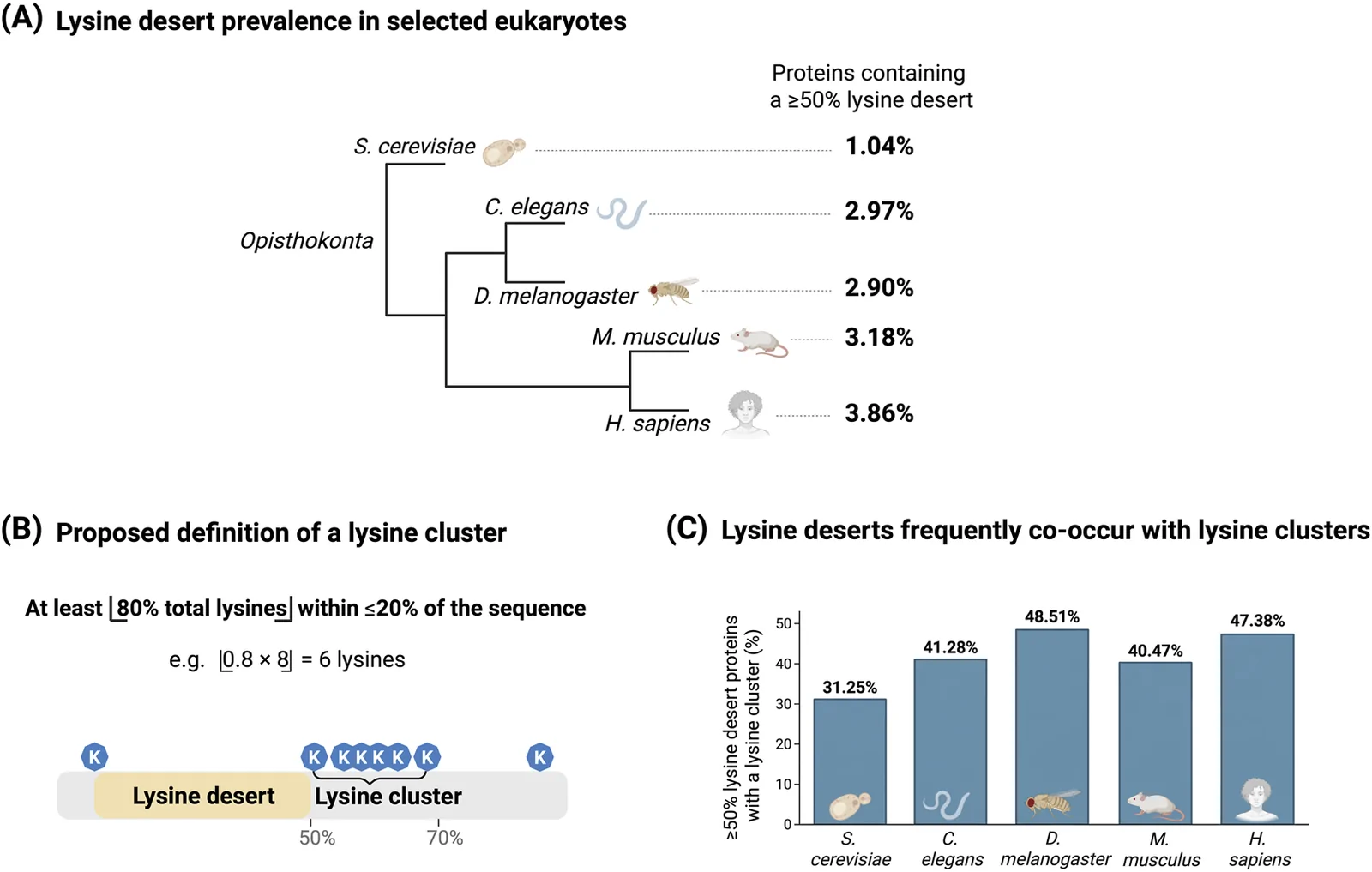
Lysine desert prevalence and co-occurrence with lysine clusters in selected eukaryotes. **(A)** Fractions of proteins containing a continuous lysine-free region spanning ≥50% of their sequence. Proteomes were filtered to include proteins of ≥150 amino acids with no more than two predicted transmembrane helices. Taxonomic relationships are schematic, and branch lengths are not to scale. **(B)** Lysine cluster criterion: at least 80% of a protein’s lysines, rounded down to the nearest integer, occur within ≤20% of its sequence; applicable only to proteins containing at least two lysines. **(C)** Fractions of lysine desert proteins that also met the lysine cluster criterion in the same filtered proteomes. Panels adapted using BioRender.com from Figures 3A, 4C,D of Szulc et al. (2023) under the Creative Commons CC BY 4.0 license.

Alongside these bioinformatic analyses, the same study examined two human non-catalytic CRL substrate receptors, VHL and SOCS1, to test whether lysine depletion could provide molecular insulation. In VHL, the lysine desert occupies a larger fraction of the mammalian protein than of its worm and fly orthologs, without a corresponding increase in protein length; at several aligned positions, lysine in the more distant orthologs corresponds to arginine in human VHL. Its three remaining lysines are exposed in the isolated protein, although CRL assembly may restrict access to one or two of them. Two VHL variants carrying distinct sets of Arg→Lys substitutions within the desert showed reduced stability, and this effect was attenuated by inhibition of neddylation, E1 activity or the proteasome. The authors next asked how the few lysines retained in native VHL influence its ubiquitylation and turnover. To address this, they compared wild-type VHL with its lysine-free variant. Both showed similar basal turnover and accumulated following proteasome inhibition. E1 inhibition also stabilized both forms, whereas neddylation inhibition moderately stabilized only wild-type VHL. NanoBRET, used by the authors as a live-cell proxy for ubiquitylation, detected similar ubiquitin-proximity signals for wild-type and lysine-free VHL, and E1 inhibition reduced the signal from both forms. Neddylation inhibition produced a less consistent reduction, reaching statistical significance in only one wild-type VHL configuration. Because NanoBRET measures proximity rather than covalent conjugation, these signals did not establish non-lysine ubiquitylation of VHL or identify the relevant acceptor sites or E3 ligases. To test whether ubiquitylated VHL-bound substrates contributed to the signal, the authors pharmacologically blocked its principal substrate-binding site. This did not reduce the NanoBRET signal, arguing against a dominant contribution from substrates bound through that site. SOCS1 showed a more pronounced difference in lysine content across vertebrates and a contrasting response to lysine restoration. Zebrafish Socs1a contains 15 lysines, whereas mouse and human SOCS1 contain only one, with arginine at many corresponding mammalian positions. The sole lysine in human SOCS1 is predicted to be buried, and three variants carrying distinct sets of Arg→Lys substitutions showed no detectable change in stability. Wild-type and lysine-free SOCS1 displayed similar basal turnover and accumulated following proteasome inhibition, but neither form was stabilized by inhibition of E1 activity or neddylation. NanoBRET detected similar ubiquitin-proximity signals for wild-type and lysine-free SOCS1, and E1 inhibition reduced the signal from both forms, whereas neddylation inhibition had no significant effect; unlike for VHL, however, no analogous substrate-binding inhibitor was available to assess the contribution of ubiquitylated SOCS1-bound proteins. The paired analysis therefore produced a clear divergence: wild-type and lysine-free forms of both receptors showed proteasome-sensitive turnover, but lysine restoration destabilized VHL without detectably affecting SOCS1 stability. Moreover, E1 inhibition stabilized both forms of VHL but neither form of SOCS1, even though the lysine-free forms of both receptors retained E1-dependent ubiquitin-proximity signals (Szulc et al., 2023). The VHL findings provide indirect support for a protective role of local lysine depletion: the introduced lysines sensitized the protein to degradation through a neddylation-dependent ubiquitin pathway, plausibly involving a CRL, although neither increased ubiquitylation of the lysine-restored variants nor modification of the introduced residues was demonstrated directly. For SOCS1, lysine introduction did not detectably affect stability, providing no support for stability-based protection under the conditions tested. Its proteasome-sensitive but E1-insensitive turnover is compatible with ubiquitin-independent degradation, while the NanoBRET signal could reflect either non-degradative ubiquitylation at a non-lysine site or proximity to a ubiquitylated partner.

To identify factors that sustain protein degradation when proteasome capacity is reduced, Yanagi and colleagues conducted an unbiased genetic screen in *Caenorhabditis elegans skn-1a* mutants (Yanagi et al., 2026). This background was chosen because SKN-1A, the worm counterpart of mammalian Nrf1, is a transcription factor that promotes proteasome biogenesis, and its loss reduces proteasome abundance. The screen identified *eel-1* mutations that increased accumulation of a ubiquitin-fusion degradation reporter. EEL-1, the worm ortholog of human HUWE1 and yeast Tom1, is a homologous to the E6-AP carboxyl terminus (HECT)-type E3 ligase with conserved lysine deserts encompassing an acidic disordered region and three ubiquitin-binding motifs. Several screen-derived mutations introduced lysine within or adjacent to these regions, leading the authors to investigate how lysine depletion contributes to EEL-1 function. Two of these lysine substitutions were tested individually in a *skn-1a* mutant background. Each substitution increased accumulation of the reporter, whereas matched alanine or arginine substitutions did not. Likewise, introducing a lysine at an arbitrarily selected position within a lysine desert impaired reporter degradation. These observations suggested that the phenotype was specifically associated with lysine introduction rather than sequence perturbation *per se*. Endogenous EEL-1 protein was undetectable in one lysine variant and barely detectable in the other. Inactivation of the HECT-domain active site markedly increased the abundance of both wild-type EEL-1 and one tested lysine variant, suggesting that autoubiquitylation regulates EEL-1 abundance and that introduced lysines enhance this process. However, neither EEL-1 ubiquitylation nor modification of the introduced lysines was demonstrated directly, and proteasome dependence of the reduction in EEL-1 abundance was not tested. The authors also examined the physiological consequences of lysine introduction in the *skn-1a* mutant background and found that the effects depended on lysine position and animal age. One substitution caused an intermediate increase in vulval rupture, another produced a delayed phenotype, and the third caused rupture comparable to that of the *eel-1* null mutant on the second day of adulthood. Finally, the authors inserted a binding tag into EEL-1 to tether a fluorescent substrate to the protein. Adding a single lysine beside the tag left more of the substrate intact, suggesting impaired degradation. In a separate rescue experiment, binding a protein to the tag reduced both accumulation of the ubiquitin-fusion degradation reporter and vulval rupture, suggesting that shielding the introduced lysine could alleviate its detrimental effects (Yanagi et al., 2026). These findings provide indirect support for molecular insulation by linking lysine introduction to ligase-dependent loss of EEL-1 abundance and functional impairment, without directly demonstrating EEL-1 ubiquitylation.

Collectively, these studies support a local rather than protein-wide effect of lysine depletion. Lysine deserts can reduce the availability of productive ubiquitin acceptor sites within particular regions without preventing ubiquitylation or degradation of the protein *per se*. Across the systems discussed above, introducing lysines within desert regions caused ligase-dependent self-destabilization, proteasome-sensitive loss of abundance, increased protein-level ubiquitylation, functional impairment or no detectable effect, depending on the protein, pathway and position examined (Figure 3). An ideal test of molecular insulation would show that an introduced, appropriately positioned lysine is directly modified by the relevant ubiquitin-transfer machinery and that this modification causes a pathway-dependent change in protein abundance or function. San1 and Slx5–Slx8 provide particularly strong evidence for protection from self-targeted degradation: San1 through extensive positional testing and sole-lysine constructs (Fredrickson et al., 2013), and Slx5–Slx8 through RING- and proteasome-dependent effects on abundance (Sharma et al., 2017). Complementary site-level evidence came from Hrd1, RAD23A and UBQLN1, in which introduced lysines were directly mapped as ubiquitin acceptors (Kampmeyer et al., 2023; Peterson et al., 2023). EEL-1 further extended the functional evidence to physiological phenotypes in a multicellular organism (Yanagi et al., 2026). However, SOCS1 showed that lysine availability is not always rate-limiting for protein stability (Szulc et al., 2023), whereas evidence for a protective role of the PSMF1 lysine desert remains inconclusive because only one lysine position was tested and no downstream outcomes were examined (Kampmeyer et al., 2023). The studies discussed here are also summarized in Table 1.

**FIGURE 3:**
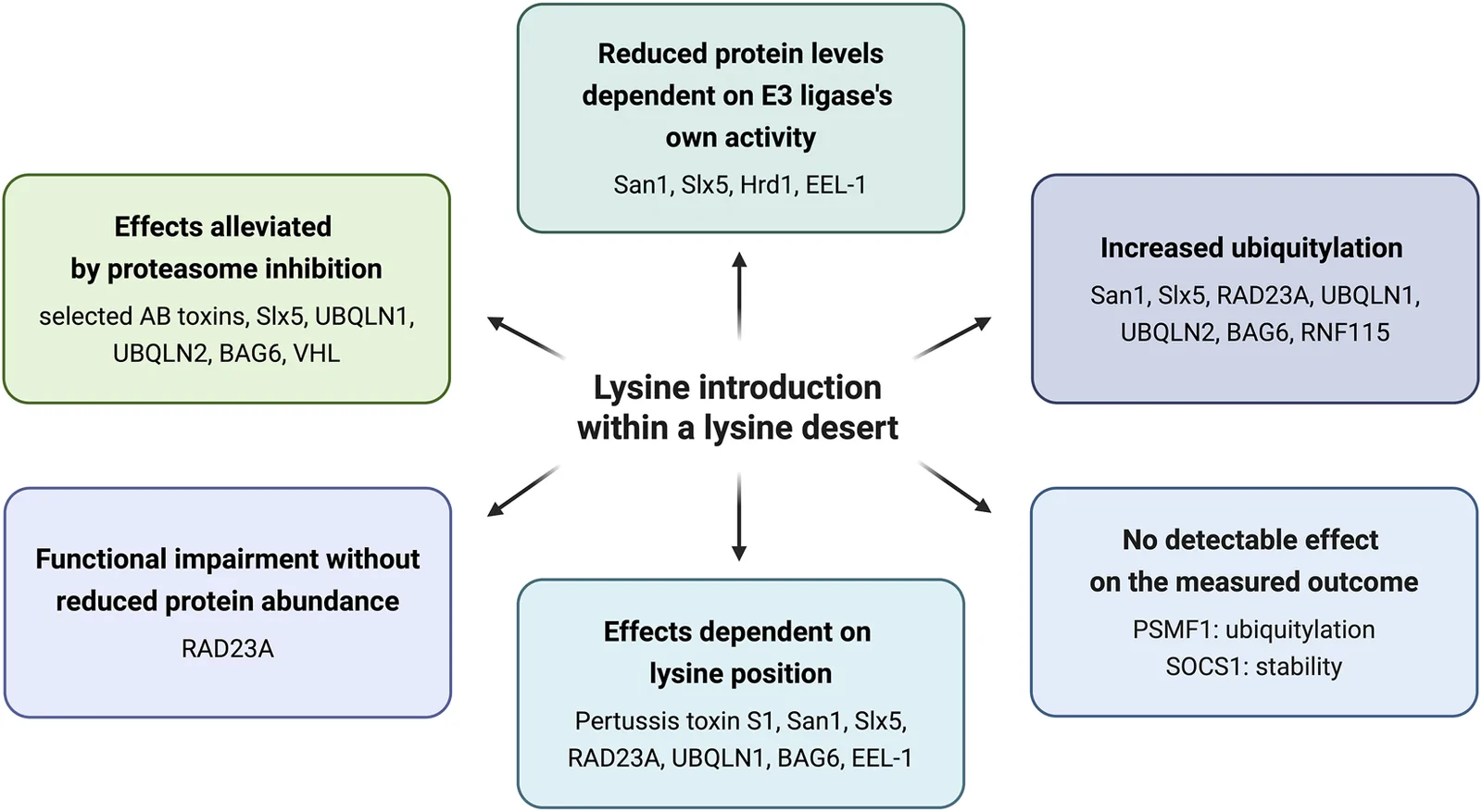
Context-dependent outcomes of lysine restoration within lysine deserts. Selected examples are shown; categories are not mutually exclusive. Created in BioRender.com.

To varying degrees, these perturbation studies established what lysine depletion can do in particular systems, but not why any given lysine desert evolved. Molecular insulation should therefore be treated as a context-dependent model that can be tested through controlled lysine-restoration experiments rather than as an intrinsic property of every lysine desert.

## 4 Alternative acceptor sites and proteolytic pathways can bypass lysine depletion

Ubiquitylation and degradation can persist after lysine loss through several mechanisms. Even when lysine ε-amino acceptors are absent, ubiquitin can potentially be attached to a free N-terminal α-amino group or to cysteine, serine or threonine side chains (Dikic and Schulman, 2023). Proteasomal degradation can also proceed without substrate ubiquitylation (Arkinson et al., 2025). Lysine depletion therefore removes a major class of ubiquitin acceptor sites without necessarily preventing ubiquitin-dependent regulation or proteasomal degradation.

Early experiments with MyoD demonstrated that its native lysines are not essential for ubiquitin-dependent degradation. Replacing all lysines with arginines approximately doubled its half-life but did not abolish polyubiquitylation or proteasome-dependent degradation. By contrast, selectively blocking the free N-terminal α-amino group prevented MyoD ubiquitylation and stabilized the protein *in vitro*, and addition of an N-terminal Myc tag stabilized it both *in vitro* and in cells (Breitschopf et al., 1998). Although the lysine-free MyoD construct was engineered rather than a natural lysine desert, these experiments provided evidence that the free N-terminus can support ubiquitin-dependent proteasomal degradation in the absence of lysines.

Studies of naturally lysine-free proteins subsequently provided further evidence for N-terminal ubiquitylation. Endogenous p16^INK4a^ was ubiquitylated and underwent proteasome-sensitive degradation in sparse cells, whereas HPV-58 E7 was polyubiquitylated in a cell-free system and degraded in a proteasome-dependent manner in transfected cells. Mass spectrometry directly identified an N-terminal ubiquitin junction in E7, whereas the acceptor site in p16^INK4a^ was not mapped (Ben-Saadon et al., 2004). Similarly, replacing the sole lysine of Epstein-Barr virus latent membrane protein 1 (LMP1) with arginine produced a lysine-free variant that remained ubiquitylated and underwent E1- and proteasome-dependent degradation. N-terminal Myc tagging stabilized both wild-type and lysine-free LMP1, while deletion of its first 12 or 24 residues stabilized the lysine-free variant (Aviel et al., 2000). These findings implicated the N-terminal region in targeting LMP1 for degradation, although ubiquitin attachment to the N-terminus was not demonstrated directly. Kuo and colleagues likewise found that naturally lysine-free human p14^ARF^ and a lysine-free variant of its mouse ortholog p19^Arf^ were polyubiquitylated and stabilized by E1 inactivation; p19^Arf^ turnover was also proteasome-sensitive. Factor Xa cleavage localized polyubiquitin to an N-terminal segment of p19^Arf^, while variants favoring N-terminal acetylation showed reduced polyubiquitylation and slower turnover (Kuo et al., 2004). Again, these results support ubiquitin attachment to the free N-terminus, although the precise junction was not mapped directly.

More recently, El Motiam and colleagues found that the N-terminal region of p14^ARF^ also supports SUMO modification. SUMO2-modified p14^ARF^ was detected *in vitro* and in cells, for both ectopically expressed and endogenous p14^ARF^. Chemical blocking of the free N-terminal α-amino group prevented detectable SUMOylation *in vitro*, while promoting N-terminal acetylation or deleting the first 35 residues eliminated the modification in cells. SUMO2 overexpression increased p14^ARF^ abundance, whereas depletion of the SUMO-conjugating enzyme UBC9 or inhibition of SUMO E1 shortened its half-life. Inhibition of ubiquitin activation or neddylation also increased p14^ARF^ SUMOylation. Together, these findings suggest crosstalk and possible competition between the pathways, although neither the SUMO acceptor site nor direct competition for the N-terminal α-amino group was demonstrated (El Motiam et al., 2025).

For ubiquitylation, the E2 enzyme UBE2W provides a defined enzymatic route when lysine acceptors are unavailable. Scaglione and colleagues showed that UBE2W preferentially monoubiquitylates protein N-termini *in vitro* (Scaglione et al., 2013). Using an inducible UBE2W-overexpression system, Davies and colleagues identified numerous putative cellular substrates. N-terminal ubiquitylation signals generally remained unchanged following proteasome inhibition, consistent with this modification not generally serving as a proteasomal degradation signal under the tested conditions. Among the identified substrates were the deubiquitylating enzymes UCHL1 and UCHL5. UBE2W monoubiquitylated lysine-free forms of both proteins *in vitro*, whereas N-terminal ubiquitin mimetics inhibited UCHL1 but enhanced UCHL5 catalytic activity (Davies et al., 2021). Thus, the free N-terminal amino group provides an alternative ubiquitin acceptor when lysines are absent, but the consequences of its modification depend on the substrate.

Beyond the N-terminus, ubiquitin can also be transferred to cysteine, serine or threonine side chains. MIR1, an E3 ubiquitin ligase encoded by Kaposi’s sarcoma-associated herpesvirus, promoted predominantly cysteine-dependent ubiquitylation and lysosomal degradation of a major histocompatibility complex class I variant lacking both cytoplasmic-tail lysines (Cadwell and Coscoy, 2005). NY-ESO-1 cancer/testis antigen naturally contains a single lysine, yet replacing it did not prevent ubiquitylation or proteasome-dependent processing, and chemical sensitivity implicated serine/threonine oxyester linkages (Golnik et al., 2016). Thus, loss of lysine acceptors need not prevent ubiquitylation, because both the free N-terminal α-amino group and nucleophilic side chains can provide alternative attachment sites. However, the prevalence and physiological importance of these routes in proteins containing natural lysine deserts remain unclear.

## 5 Lysine desert enrichment in Actinobacteria and their phages raises a testable Pup-proteasome adaptation hypothesis

Lysine-dependent proteolytic tagging also occurs beyond the eukaryotic ubiquitin system. Many Actinobacteria possess the Pup-proteasome system (PPS), which is functionally analogous to the UPS but chemically distinct. The small, intrinsically disordered prokaryotic ubiquitin-like protein (Pup) is conjugated by PafA ligase through its C-terminal glutamate to the ε-amino group of a substrate lysine. The Pup-tagged substrate can then be engaged by the Mpa/ARC ATPase and translocated into the 20S proteasome. Because pupylation does not employ the E1–E2–E3 cascade, the PPS represents an independently evolved system in which lysine availability may influence access to a proteolytic tag (Barandun et al., 2012).

A systematic analysis surveyed more than 8,000 bacterial reference proteomes using two lysine desert definitions: an absolute one requiring a continuous lysine-free region of ≥150 residues and a relative one requiring such a region to span ≥50% of the protein sequence. Under both criteria, Actinobacteria showed the highest average prevalence of lysine desert proteins; results obtained using the relative definition are shown in Figure 4A. Among the selected Actinobacteria, lysine deserts were more frequent in species possessing both pupylation and proteasomal machinery (*Streptomyces coelicolor*, *Mycobacterium tuberculosis, Mycolicibacterium smegmatis*) than in *Corynebacterium glutamicum*, which can pupylate proteins but lacks the proteasomal subunits (Figure 4B). Among *M. tuberculosis*, *M. smegmatis* and *C. glutamicum*, lysine deserts were also detected in proteins reported as pupylated, with the highest fraction observed in *M. tuberculosis*. After these reported substrates were excluded, lysine deserts remained more frequent in the two species possessing both pupylation and proteasomal machinery. However, the available pupylome datasets include only approximately 1% of the respective proteomes, so proteins absent from these datasets cannot reliably be classified as non-substrates of the PPS (Szulc et al., 2023).

**FIGURE 4:**
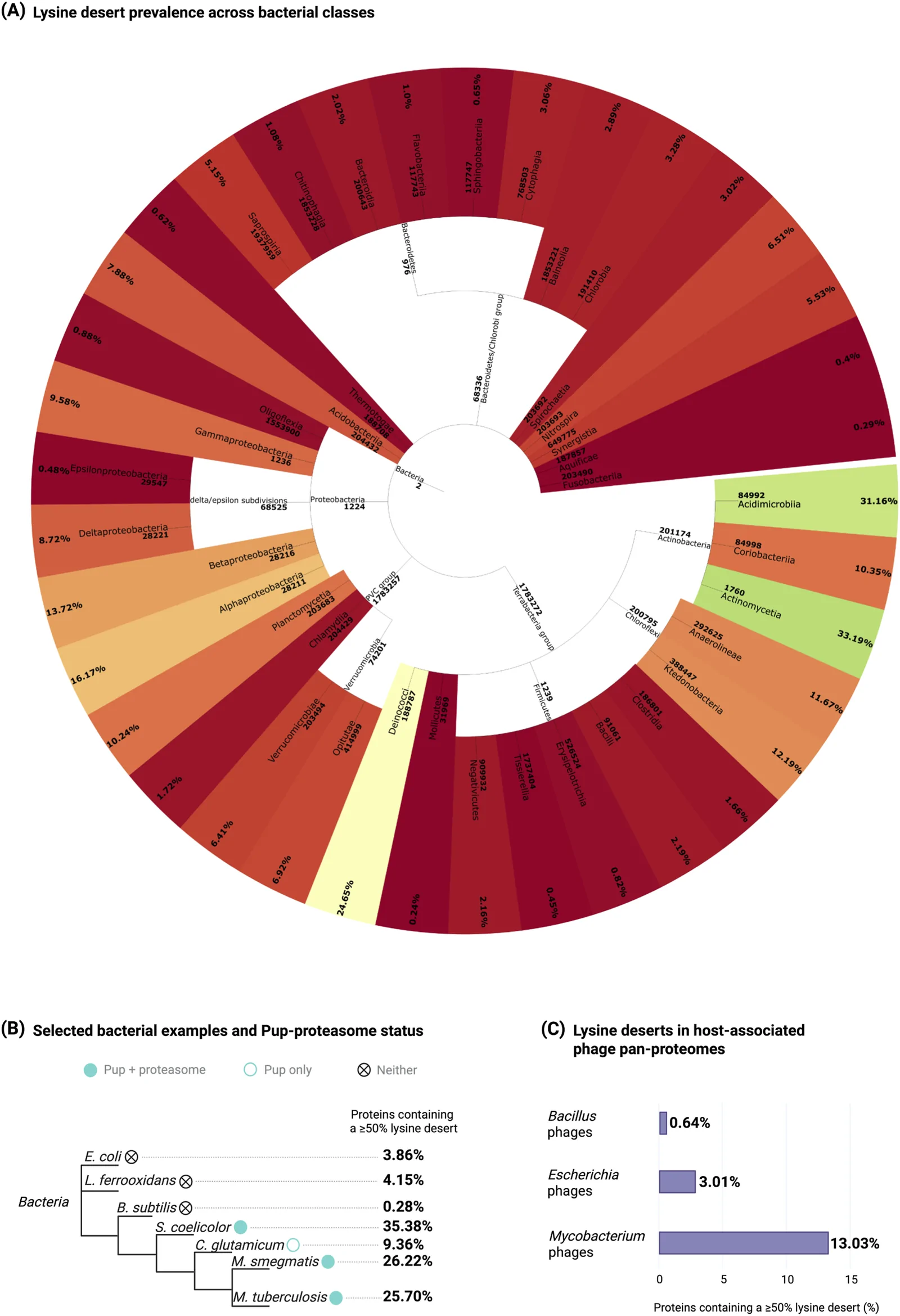
Lysine desert prevalence in bacteria and host-associated phages. **(A)** Class-level mean fractions of proteins containing a continuous lysine-free region spanning ≥50% of the sequence across 8,881 bacterial reference proteomes. Only classes represented by at least ten taxa are shown; color indicates min–max-normalized prevalence, from the lowest values in red to the highest values in green. **(B)** Lysine desert prevalence and Pup–proteasome status in seven selected bacterial species. Taxonomic relationships are schematic, and branch lengths are not to scale. **(C)** Fractions of proteins containing a continuous lysine-free region spanning ≥50% of the sequence in non-redundant pan-proteomes constructed from 133 to 527 phages associated with *Bacillus*, *Escherichia* or *Mycobacterium*. The analyses in panels A–C were restricted to proteins of at least 150 amino acids; panel B additionally excluded proteins with more than two predicted transmembrane helices. Panel A is reproduced, and panels B and C were adapted using BioRender.com, from Figure 2 of Szulc et al. (2023) under the Creative Commons CC BY 4.0 license.

A parallel pattern emerged from three non-redundant phage pan-proteomes, each assembled from 133 to 527 phage proteomes associated with a particular host genus. Under the relative criterion, lysine desert proteins were approximately fourfold more frequent in the *Mycobacterium* phage pan-proteome than in the *Escherichia* phage pan-proteome and more than 20-fold more frequent than in the *Bacillus* phage pan-proteome (Figure 4C); the absolute 150-residue criterion produced the same ordering (Szulc et al., 2023). This host-associated pattern is consistent with the possibility that exposure to the host PPS favors lysine depletion in *Mycobacterium* phage proteins, potentially limiting their pupylation during infection. This adaptive interpretation remains hypothetical, however, because genomic GC content and the distribution of pupylation and proteasomal machinery have not been evaluated jointly while accounting for phylogenetic non-independence.

A major alternative explanation for lysine depletion in Actinobacteria is genomic GC bias. Lysine is encoded by the A-rich codons AAA and AAG, whereas four of the six arginine codons are GC-rich; increasing genomic GC content can therefore reduce lysine use while favoring arginine (Lightfield et al., 2011; Barceló-Antemate et al., 2023). Halophilic archaea illustrate how strongly this bias can influence protein composition. Fukuchi and colleagues found that the reduced lysine abundance in halophilic proteins was close to that expected from genomic composition and proposed that it arose largely from exceptionally high GC content (Fukuchi et al., 2003). Tadeo and colleagues addressed a distinct question: whether reducing the number of surface lysines can benefit proteins under hypersaline conditions. Substituting these residues with arginines or smaller polar residues in a mesophilic model protein increased salt-dependent stabilization, whereas introducing lysines into a halophilic protein reduced its stability at high salt. These effects correlated more closely with side-chain size and solvent-accessible surface area than with net protein charge (Tadeo et al., 2009). Thus, genome-driven compositional bias and protein-level adaptation are not mutually exclusive: GC-rich genomes may favor lysine depletion, while the resulting composition may also provide a biophysical advantage in a particular environment.

Beyond genomic composition, lysine desert enrichment might reflect proteolytic pressures extending beyond the PPS. In addition to this system, mycobacteria possess bacterial-type ATP-dependent proteases, including Clp and FtsH, while Lon occurs in some species (Thomas and Dougan, 2017). Host proteases can degrade phage proteins and thus restrict infection, while phage-encoded proteins can counter this pressure by inhibiting host proteases (Holtappels et al., 2023; Beggs and Bassler, 2024). Whether a comparable evolutionary conflict contributes to the observed lysine depletion in Actinobacteria or their phages remains unknown. Unlike these proteases, however, PafA requires a substrate lysine for Pup conjugation (Barandun et al., 2012). Lysine depletion could therefore directly reduce susceptibility to pupylation, whereas it is not a recognized general strategy for evading other bacterial proteolytic systems. However, the observed associations do not establish whether PPS exposure, rather than broader proteolytic pressures or another correlated factor, caused lysine desert enrichment.

Taken together, these observations leave the enrichment of lysine deserts in Actinobacteria and their phages intriguing but unresolved. Genomic GC bias probably contributes, yet other relatively high-GC groups, including Acidobacteria and several proteobacterial classes, contained substantially fewer lysine desert proteins (Figure 4A; Szulc et al., 2023). Evaluating the PPS-adaptation hypothesis would first require a phylogenetically informed analysis testing whether lysine desert enrichment remains associated with the complete PPS after accounting for genomic composition, shared ancestry and environmental factors. Persistence of this association would strengthen the rationale for functional validation, which could begin by introducing lysines at selected positions in native actinobacterial or phage proteins and testing whether they become pupylation sites that promote degradation. For phage proteins, viral fitness could then be compared across isogenic hosts with intact or disrupted PPS components, such as Δ*pafA* or Δ*mpa* strains, and hosts in which other proteases are deleted or conditionally inhibited. If PPS disruption alleviated the fitness cost of lysine introduction whereas perturbing other proteolytic systems did not, this would support a pupylation-specific effect. Until such evidence is available, adaptation to the PPS remains a compelling but experimentally untested hypothesis for lysine desert enrichment in Actinobacteria and their phages.

## 6 Experimental design determines what lysine-restoration studies can establish

Experimental analysis of lysine deserts presents several challenges because altering lysine availability can affect protein properties beyond covalent modification. Mutational studies must therefore distinguish changes in acceptor availability from other consequences of changing residue identity. Arg→Lys introduces a potential ubiquitin acceptor, whereas Lys→Arg removes one, but both exchange chemically distinct side chains. Although lysine and arginine carry the same positive charge, the lysine side-chain amine and the planar, charge-delocalized guanidinium group of arginine support different non-covalent interactions, as illustrated by the distinct phase behavior of lysine- and arginine-rich systems (Wang et al., 2018; Fisher and Elbaum-Garfinkle, 2020). Arg→Gln controls test the effect of arginine loss without introducing lysine, but they also remove positive charge. This ambiguity was evident in UBQLN1 and UBQLN2, where the non-lysine controls also altered cytosolic organization and, in UBQLN2, abundance and ubiquitylation (Kampmeyer et al., 2023; Grønbæk-Thygesen et al., 2026). No single substitution is therefore neutral, and reversing a candidate evolutionary Lys→Arg change neither reconstructs the ancestral context nor demonstrates adaptive lysine loss. Strong experimental designs should thus compare several position-matched variants at similar abundance and localization and connect phenotypes to changes in ubiquitylation through acceptor-site mapping, pathway-specific perturbation and rescue.

Experimental readouts must also be interpreted according to what they directly measure. Ubiquitin signals detected by proximity assays or non-denaturing pull-downs may originate from the protein of interest or an associated molecule. Denaturing isolation can attribute a conjugate to the protein itself but does not identify the acceptor residue or linkage chemistry. Stabilization following E1 inhibition supports a requirement for ubiquitin activation, whereas a measured slowing of turnover following proteasome inhibition supports proteasomal degradation; an increase in steady-state abundance alone provides indirect evidence. None of these findings alone demonstrates direct ubiquitylation of the substrate. Changes in abundance may also produce secondary effects on localization, complex assembly or condensate formation. Robust mechanistic conclusions should therefore be supported by direct turnover measurements with identification of the relevant E3 ligase and, where feasible, demonstration of direct modification and identification of the acceptor sites, followed by validation in an endogenous or minimally perturbed setting.

Protein tags create an additional interpretive complication in lysine-restoration studies. Green fluorescent protein (GFP) and related tags introduce additional lysines, folded surfaces and terminal or junctional sequences that may alter the modification, stability or behavior of the tagged protein. Nevertheless, experiments with RAD23A, UBQLN1 and BAG6 showed that local acceptor position can outweigh total lysine number. Although GFP supplied multiple lysines to every construct, restoring lysines within the native deserts destabilized UBQLN1 and BAG6 but not RAD23A (Kampmeyer et al., 2023). Tagged constructs can therefore facilitate research on lysine desert proteins, but their potential contributions to ubiquitylation and protein fate must be considered.

## 7 Conclusion and perspectives

Available evidence supports lysine deserts as local, position- and geometry-dependent constraints on lysine-directed ubiquitylation rather than as general determinants of protein stability. Introducing lysines within lysine-depleted regions can promote ubiquitylation, ligase-dependent self-destabilization, proteasome-sensitive loss of abundance or functional impairment, but can also have no detectable effect. Early studies of AB toxins first showed that introducing lysines into lysine-deficient catalytic subunits could cause a proteasome-sensitive reduction in toxin activity (Deeks et al., 2002; Rodighiero et al., 2002; Worthington and Carbonetti, 2007). Studies of the yeast E3 ligases San1 and Slx5 linked lysine restoration within depleted regions to ligase-dependent autoubiquitylation and degradation, supporting local insulation from self-modification (Fredrickson et al., 2013; Sharma et al., 2017). Protein-level autoubiquitylation was demonstrated for the lysine-restored yeast E3 ligase Hrd1, and lysine introduction increased protein-level ubiquitylation of UBQLN1 and RAD23A; introduced lysines were mapped as ubiquitin acceptors in all three systems. Lysine restoration reduced Hrd1 and UBQLN1 abundance in a ligase-activity- and proteasome-dependent manner, respectively, whereas RAD23A abundance remained unaffected despite an associated functional defect (Kampmeyer et al., 2023; Peterson et al., 2023). In BAG6, UBQLN2 and RNF115, lysine restoration increased protein-level ubiquitylation; BAG6 and UBQLN2 additionally showed proteasome-sensitive reductions in abundance, whereas downstream consequences were not examined for RNF115 (Kampmeyer et al., 2023; Grønbæk-Thygesen et al., 2026). Evidence from VHL came instead from pathway perturbations: lysine-restored variants showed reduced stability, and this effect was attenuated by inhibition of neddylation, E1 activity or the proteasome, although neither increased ubiquitylation nor modification of the introduced lysines was demonstrated. SOCS1 produced a contrasting result, as three distinct lysine-restored variants showed no detectable change in stability (Szulc et al., 2023). Finally, lysine introduction into *C. elegans* EEL-1 reduced its abundance and function and caused position- and age-dependent physiological defects; catalytic inactivation restored the abundance of one tested lysine variant, although EEL-1 ubiquitylation and proteasome dependence were not demonstrated (Yanagi et al., 2026). Consistent with these context-dependent outcomes, lysine desert extent did not correlate with protein half-life across the examined human turnover datasets (Szulc et al., 2023). Table 1 summarizes the evidence from the experimental systems discussed above, while Figure 3 highlights their context-dependent outcomes.

Although experimentally studied lysine deserts occur in proteins from yeast, nematodes and humans, whether selection to limit ubiquitin attachment shaped their evolution across eukaryotes remains unresolved. The broad increase in lysine desert prevalence reported across selected model proteomes from yeast through invertebrates to mammals (Figure 2A; Szulc et al., 2023) motivates testing whether expansion and diversification of the ubiquitin system increased selective pressure for local protection from ubiquitylation. Dense sampling of orthologs could reconstruct independent lysine gains and losses and test whether desert emergence repeatedly coincides with changes in the relevant E3 ligases, interaction networks or degradation pathways after accounting for protein length, local composition, intrinsic disorder and domain architecture. Candidates supported by these analyses could then be examined by recreating inferred ancestral and derived states in their corresponding protein backgrounds and measuring modification, turnover, function and fitness. Evidence that a particular desert evolved under selection for its protective role would be stronger if restoring inferred ancestral lysines caused a functional or fitness cost that was rescued by disrupting the relevant ligase or pathway. The importance of distinguishing present-day cost from evolutionary selection is illustrated by spurious tyrosine phosphorylation in yeast: although the number of sites correlated with reduced growth and many were predicted to be deleterious, many others had negligible fitness effects, and comparative analyses found no evidence of broad counter-selection against tyrosine in species possessing tyrosine kinases (Bradley et al., 2024). Such analyses should also consider selective pressures arising from other lysine-directed modifications (Lee et al., 2023), because adaptive avoidance of modifications such as acetylation, methylation or SUMOylation remains largely unexplored. Integrating phylogenetic reconstruction with mechanistic and fitness experiments will therefore be necessary to distinguish an evolved protective function from a present-day consequence of lysine scarcity.

Interpreting the consequences of lysine depletion also requires considering alternative ubiquitin acceptors, because ubiquitin can be attached to the free N-terminal α-amino group or to cysteine, serine or threonine side chains (Dikic and Schulman, 2023). Notably, cysteine depletion can coincide with lysine deserts. In Hrd1, cysteine introductions within two such regions were associated with autoubiquitylation and reduced protein abundance, although these residues were not mapped as ubiquitin acceptors (Peterson et al., 2023). UBQLN1 and UBQLN2 contain no cysteines, raising the possibility that combined lysine and cysteine depletion could further restrict acceptor availability, but this has not been tested experimentally (Grønbæk-Thygesen et al., 2026). The extent to which natural lysine desert proteins use alternative ubiquitin acceptors therefore remains an important unresolved question. Addressing it, however, presents challenges at several stages: conventional di-glycine enrichment is directed primarily toward lysine-linked peptides, reducing conditions can disrupt thioester conjugates, and heating or alkaline conditions can hydrolyze oxyester linkages. Moreover, database searches must explicitly consider non-lysine acceptors to avoid leaving the corresponding spectra unidentified (Kelsall, 2022; van Overbeek et al., 2024). Although dedicated enrichment methods have enabled proteome-scale detection of N-terminal ubiquitylation (Akimov et al., 2018; Davies et al., 2021), determining whether lysine deserts redirect modification to cysteine, serine or threonine will require new methodologies combining linkage-preserving sample preparation, enrichment strategies not restricted to lysine-linked remnants and spectral searches configured to identify non-lysine acceptors.

Regional lysine depletion also draws attention to lysines retained elsewhere in the same protein. Lysine clusters frequently coexist with lysine deserts (Figure 2C) and tend to occur in more structured regions, including the RING domains of several E3 ligases (Szulc et al., 2023). UBQLN2 provides a suggestive example of such regional partitioning: native ubiquitin acceptors were mapped within its N-terminal ubiquitin-like domain, whereas lysines introduced into its C-terminal desert sensitized the protein to E6AP-dependent proteasomal degradation. E6AP disruption, however, only modestly increased wild-type abundance and did not alter its protein-level ubiquitylation (Grønbæk-Thygesen et al., 2026). Together, these observations raise the possibility that lysine depletion and retention in different regions jointly partition ubiquitylation and its consequences within the same protein, extending the local molecular insulation model.

Future studies could use lysine deserts as test cases for improving the prediction and design of targeted protein degradation, with distinct objectives for the target and the recruited E3 ligase. On the target side, lysine desert proteins could help reveal how local sequence, protein conformation and degrader-imposed orientation determine whether available lysines become productive ubiquitin acceptors. This knowledge could guide the selection of target-binding sites and linker geometries that present otherwise poorly positioned lysines to the ubiquitin-transfer machinery (Smith et al., 2019; Wurz et al., 2023). On the E3 side, the converse objective would be to maintain efficient ubiquitin transfer to the target while limiting ligase self-ubiquitylation and turnover. Degrader-induced geometry might therefore be tuned to shield or unfavorably orient lysines within the recruited E3, potentially prolonging its availability. This possibility is already relevant to VHL, a lysine desert substrate receptor within one of the most widely used E3 systems in the development of proteolysis-targeting chimeras (Urbina et al., 2025), and may acquire broader importance as other lysine desert E3 ligases become accessible to chemical recruitment. Engineered degradation of proteins lacking suitable lysine acceptors provides another potential application. In a multivalent Trim-Away system, a lysine-free substrate was ubiquitylated and degraded using a lysine-free RING–nanobody ligase, demonstrating that enforced recruitment and clustering can bypass lysine acceptors (Kiss et al., 2023). Identifying the alternative acceptor sites and linkage chemistry responsible could inform strategies for degrading naturally lysine-scarce proteins or targets whose lysines are poorly positioned for ubiquitin transfer. Systematic comparisons across E3–target geometries could therefore determine how degrader design might promote productive target ubiquitylation, limit self-ubiquitylation and turnover of the recruited E3, and exploit alternative acceptors when target lysines are scarce or poorly positioned.
